# Rift Valley Fever in Rwanda Is Urging for Enhancing Global Health Security Through Multisectoral One Health Strategy

**DOI:** 10.3390/microorganisms13010091

**Published:** 2025-01-05

**Authors:** Claude Mambo Muvunyi, Jean Claude Semuto Ngabonziza, Emmanuel Edwar Siddig, Ayman Ahmed

**Affiliations:** 1 Rwanda Biomedical Centre, Kigali 7162, Rwanda; 2 Department of Clinical Biology, University of Rwanda, Kigali 3900, Rwanda; 3 Research, Innovation and Data Science Division, Rwanda Biomedical Centre, Kigali 7162, Rwanda; 4 The Africa Centres for Disease Control and Prevention (Africa CDC), Ring Road, 16/17, Haile Garment Lafto Square, Addis Ababa P.O. Box 3243, Ethiopia; 5 Pan-Africa One Health Institute (PAOHI), Kigali 11KG ST203, Rwanda

**Keywords:** emerging zoonotic diseases outbreaks, arboviral diseases, haemorrhagichemorrhagic fevers, livestock health and productivity, food insecurity, mosquito vectors, socioeconomic impacts, climate change, global health security, pandemic preparedness and response, Africa

## Abstract

Rift Valley fever (RVF) is a devastating zoonotic mosquito-borne viral hemorrhagic fever disease that threats human and animal health and biodiversity in Africa, including in Rwanda. RVF is increasingly outbreaking in Africa, leading to devastating impacts on health, socioeconomic stability and growth, and food insecurity in the region, particularly among livestock-dependent communi-ties. This systematic review synthesizes existing evidence on RVF’s epidemiology, transmission dynamics, and the prevention and control measures implemented in Rwanda. Our findings high-light the rapidly increasing prevalence of RVF and the expansion of its geographical distribution and host range in Rwanda. Furthermore, the review reveals gaps in local evidence, including the existence of competent vectors of RVFV and the risk factors associated with the emergence and spread of RVF in the country. This underscores the urgent need for prospective research to inform evidence-based health policymaking, strategic planning, and the development and implementation of cost-effective preventive and control measures, including diagnosis and surveillance for early detection and response. It also calls for the institutionalization of a cost-effective, multisectoral, and transdisciplinary One Health strategy for reducing the burden and risk of climate climate-sensitive and zoonotic diseases, including RVF, in the country. We recommend exploring cost-effective human and/or animal vaccination mechanisms for RVF, integrating AI-powered drones into dis-ease vectors surveillance and control, and the routine implementation of genomics-enhanced xenosurveillance to monitor changes in pathogens and vectors dynamics in order to inform poli-cymaking and guide the control interventions.

## 1. Introduction

Rift Valley fever (RVF) is an emerging zoonotic viral haemorrhagichemorrhagic fever disease caused by the Rift Valley fever virus (RVFV), which belongs to the genus Phlebovirus in the family Phenuiviridae [1]. The RVFV is a mosquito-borne zoonotic vi-rus that readily infects both human and animal populations, as well as hematophagous arthropods in the environment; hence, it is known as an arthropod-borne viral (arbo-viral) disease. The virus emerges periodically, causing epizootics among animals, epi-demics among humans, and sometimes significant outbreaks that involve both human and animal populations in the area [2,3]. In addition to several competent mosquito vectors, the RVFV is also transmitted by through close contact with infected animals and their products, such as the consumption of raw milk and uncooked meat from in-fected animals, as well as vertical transmission from mother to child in human and animal populations [4,5]. Additionally, the transmission cycle of the RVFV in the area is mainly sustained through transovarial vertical transmission from a mother to her offspring among vector populations; particularly the Aedes vexan mosquito, due to its unique bionomics, including its lifecycle [6]. Furthermore, the wide range of suscepti-ble hosts of RVF supports the continuation of the RVFV transmission and its sporadic emergence in endemic areas [7]. Moreover, the uncontrolled movement of infected hosts, including human, livestock, wildlife, and vector populations, is a driving force for the spread, emergence, and steady expansion of the geographical distribution of the disease [3,8,9]. 

Most infections with the RVFV are mild or asymptomatic; however, the case fatal-ity rate for the disease is over 10%, and this is increased among vulnerable popula-tions, including young children and pregnant women [10]. Moreover, the disease is associated with high mortality and abortion rates among livestock that could becan reach up to 100%, resulting in the wiping out of herds, which eventually leads to a se-vere socioeconomic burden among livestock-dependent communities [10,11,12]. There-fore, RVF is on lists by the World Health Organization (WHO), the Global Alliance for Vaccines and Immunizations (GAVI), and the Coalition for Epidemic Preparedness Innovations (CEPI) lists for high-priority diseases that might cause the next pandemic [13,14,15,16,17]. 

Several risk factors, including globalization, unplanned urbanization, and chang-es in the climate, land use, and land cover, are driving a rapid shift in the dynamics of human, animal, and disease vector populations [18,19]. This is indicated by the in-creasing rate of disease emergence and outbreaks development globally and in the re-gion, including Rwanda, which is seeing a steadily growing burden of emerging infec-tious diseases, including vector-borne arboviral diseases like RVF but also Zika, Den-gue, and Chikungunya [3,20,21], and along with other emerging zoonotic diseases such as mpox and, more recently, Marburg virus disease [22,23,24]. Like most African coun-tries, this increasing emergence rate and, prevalence of diseases, and along with the expansion of their host ranges and the geographical distributions, of diseases are fuelledfueled by several interconnected risk factors. These risk factors include changes in the climate, living environments, land use, land cover, and the dynamics of human, animal, and vector populations [25,26,27,28]. 

In Rwanda, RVF is increasingly becoming an issue for the public health and socio-economic stability in the country, due to its growing rates and the sizes of disease out-breaks that have affected both human and livestock populations in the country over the recent years [3,29,30]. It This has emerged as a significant public health and veter-inary challenge, particularly in regions where livestock farming is a vital part of the economy [31]. 

Nevertheless, despite the increasing growth in RVF burden and its prevalence in the country, there is still a lack of comprehensive analyses of the existing evidence on the disease in the this country [3]. Therefore, this review aims to synthesize the availa-ble evidence on the disease in the country; provide ans in-depth analysis of the current situation; explores future perspectives; summarizes the current state of knowledge; and determines gaps in health policies, planning, and the implementation of preven-tion and control measures. The objective of this analysis is to inform future innovative policymaking, strategic planning, and, resources mobilization, and guide the develop-ment and implementation of innovative, cost-effective, and sustainable interventions for the surveillance and control of RVF in Rwanda.

## 2. Materials and Methods

In this study, we performed a systematic literature review on RVF in Rwanda, following the up-to-date Preferred Reporting Items for Systematic Reviews and Meta-Analyses (PRISMA) guidelines in data mining and the identification, screening, and inclusion of relevant studies and reports [32,33]. Our systematic review was not registered; however, we developed and implemented the following protocol.

In brief, to ensure comprehensive data collection, we searched both peer-reviewed and gray literature sources without any time or language restrictions; this search was updated in December 2024. The search terms “Rift Valley fever Rwanda” were used across multiple scientific databases, including PubMed and Google Scholar. Additionally, we reviewed official reports available on the websites of Rwandan authorities for human and animal health, the WHO for human health-related reports, and the intergovernmental World Organization for Animal Health (WOAH) for animal health reports. We also consulted the websites of other relevant organizations and stakeholders involved in RVF research and control. Moreover, we thoroughly checked the reference lists of the identified reports for additional resources that contained relevant information about RVF in Rwanda.

The data search and identification, screening, and decision of which reports to include in our study were independently performed by two co-authors. In the case of a discrepancy or disagreement, a third and/or fourth author was consulted on a case-by-case basis to double-check the decision independently and make the final decision, ensuring consistency and accuracy in data selection.

Furthermore, the main eligibility criteria for reports to be included in this review were that they contained data about the disease’s epidemiology and prevention and control measures that have been implemented in Rwanda. Relevant data were extracted into Excel spreadsheets and synthesized through analysis and mapping into evidence that mainly represented prevalence, geographical distribution, host range, date, implemented interventions, and gaps in policies and implementation. Synthesized data were tabulated and visualized through illustrations and maps

## 3. Results

**Figure 1:**
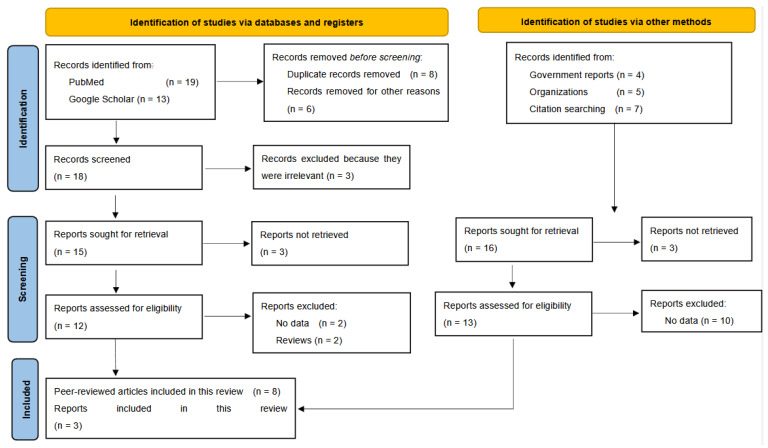
PRISMA flowchart summarizing the systematic data mining, identification and screening of records, and final inclusion of reports.

Our systematic data search and mining for peer-reviewed articles and gray literature that included relevant information about RVF in Rwanda initially identified 32 peer-reviewed articles and 4 Government reports (Figure 1). Additionally, five reports were identified in international organizations’ databases, alongside seven reports that were retrieved from citations (Figure 1). These reports included eight peer-reviewed articles and three gray literature reports (Figure 1). However, only eight articles and three reports met the eligibility criteria for inclusion in this review, as they included information about the emergence, epidemiology, geographical distribution, host range, and genomic characterization of the RVFV, as well as prevention and response measures for RVF outbreaks in the country (Figure 1) [3,29,30,31,34,35,36,37,38,39,40]. 

The first emergence of the RVFV in Rwanda was confirmed in 2011, when the virus was involved in an epizootic among cattle [30,39]. Later, serological investigations revealed a high seroprevalence rate of 17% for the RVFV, with the highest exposure rate of 37% detected among cattle in the Kirehe district [30]. Then, no exposure to or cases of the RVFV were detected and/or reported among humans or animals in Rwanda for the next five years. During this time, the country was assumed to be RVF-free. 

However, RVF re-emerged in the country, leading to the development of an epizootic that affected livestock populations across the country in 2018 [30,39]. In this epizootic of RVF, 44 cattle and 12 goats tested positive for the RVFV by RT-PCR. Although no infections were reported among human or sheep populations, two veterinarians who were caring for sick animals were believed to have had RVF [40].

In 2022, a larger outbreak of RVF occurred, and this affected both human and livestock populations across the country [3,31]. This outbreak was substantially larger compared to the previous epizootic with regard to infections among humans and animals. It involved over 3,000 livestock infections, including cattle, goat, and sheep [31]. Additionally, this outbreak spread throughout the country and resulted in about 170 human infections and 22 deaths [3,29].

Eight reports contained quantitative data on RVF’s epidemiology among human and animal populations in Rwanda; these reports are synthesized and summarized in Table 1 [3,29,30,31,34,39,40,41]. Alarmingly, these reports indicate a steadily growing burden of RVF in the country, with a reported prevalence of up to 100% among tested populations (Table 1). 

The other studies that we synthesized include reports on the genomic analysis of the RVFV [35], community engagement and involvement in RVF prevention and control [36], and the innovative integration of advanced technology, namely the use of drones for the timely delivery of an RVF vaccine to livestock in remote and difficult-to-reach areas throughout the country [38]. 

**Figure 2:**
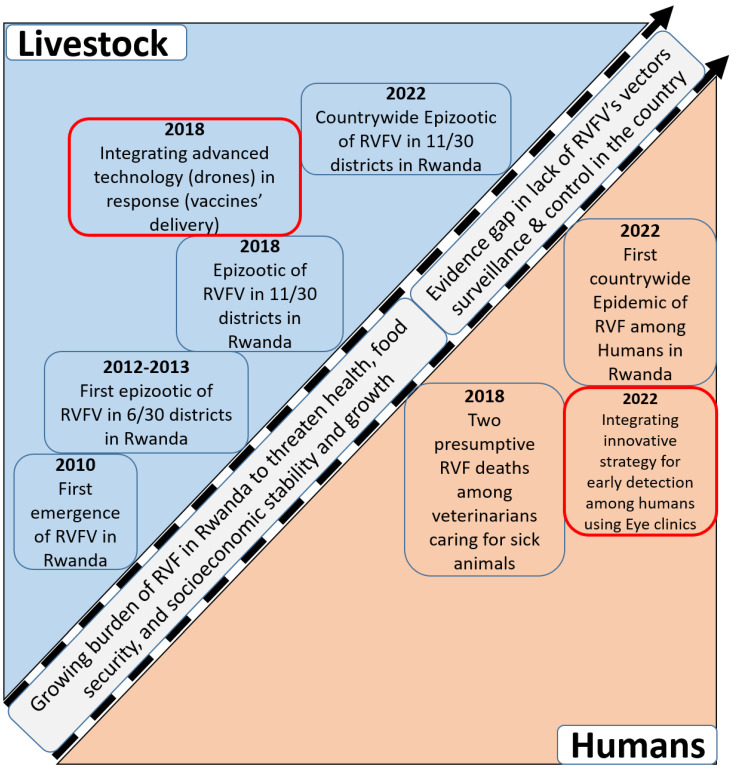
Illustration of the development of the Rift Valley fever burden in Rwanda; aspects related to the integration of innovative strategies for early detection and response to outbreaks are highlighted in red boxes, and gaps in evidence and policymaking are presented in the diagonal boxes.

Regarding policy gaps and the need for evidence, despite the steadily growing burden of RVF in the country, there is a persisting lack of evidence about the composition and distribution of the RVFV’s vectors in the country (Figure 2). This, in turn, has resulted in gaps in policymaking, planning, and the cost-effective implementation of vector surveillance and control in the country (Figure 2). 

**Figure 3:**
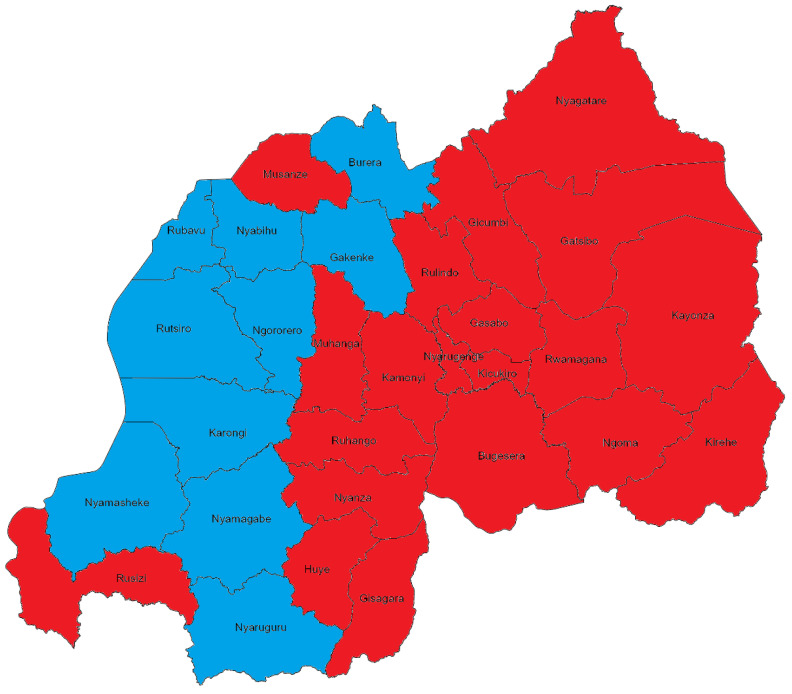
Map of Rwanda showing the distribution of RVF infections among human and livestock populations in districts highlighted in red. Meanwhile, districts that only reported infections among livestock without the involvement of humans are highlighted in blue.

The geographical distribution of the RVFV’s infections in Rwanda via hosts is rapidly growing; this is indicated by the rapid growth in human infections with the RVFV, with an increase from 0 to 66% (20/30) of districts in the country having reported infections/deaths due to the RVFV by 2022 (Figure 3). Meanwhile, infections with the RVFV among livestock had been reported throughout the entire country by 2022 (Figure 3).

Human cases of RVF reported in 2022 stemmed from the Nyagatare district, where RVF was steadily reported among livestock, which indicates the zoonotic origin and spillover of RVF. Therefore, this highlights the importance of raising awareness among at-risk communities, mainly including the veterinary community, farmers, communities that consume raw milk and uncooked meat, and public health practitioners to self-implement preventive measures [31]. The devastating impacts on livestock, evidenced by over 3,000 affected animals, representing 0.1% of the country’s livestock resources, and the staggering number of deaths (516) and abortions (1254) that occurred in 2022, underscore the socioeconomic and food insecurity ramifications of RVF in the country [31]. This represents the substantial growth in the burden and impacts of RVF in the country since the previous outbreak in 2018, which was mainly clustered in the Eastern part of the country, seemingly as an extension of the regional outbreaks of RVF that concurrently occurred in Kenya, Uganda, and Tanzania [40]. Nevertheless, the RVF outbreak in 2018 only involved the deaths of two veterinarians presumed to be caused by RVF, who died while caring for livestock sick with RVF [40]; however, their infection with the RVFV was not confirmed through laboratory tests. On the other hand, the RVF outbreak in 2022 was nationwide and involved 173 and 22 human infections and deaths, respectively, suggesting that the disease was becoming established and endemic in the country. This is further supported by the expansion of the geographical distribution and range of hosts involved in the disease’s dynamics in the country [3]. Genomic similarity and an evolutionary linkage were established between the virus isolates from the two outbreaks in Rwanda and they were confirmed to be associated with the same viral lineage as RVF outbreaks in Uganda [35]. 

Unfortunately, there was not a single study that reported on RVF’s vectors in Rwanda. This highlights a significant gap in evidence on the vector composition and distributions. Accordingly, this lack of data on vectors has a direct impact on the development and implementation of evidence-based cost-effective interventions for the preparedness against and prevention and control of RVF in the country. Nevertheless, interventions that were implemented in the country include vaccinating at-risk livestock and community engagement aimed at improving community awareness to enhance preparedness, prevention, and early detection and response through community-led syndromic surveillance throughout the country [31,36,37,38]. Additionally, health education and awareness-raising activities targeting farmers and livestock owners were effective strategies for engaging at-risk and affected communities in the surveillance, early detection, and prevention of and response to outbreaks of the disease [36].

## 4. Discussion

Our analysis revealed an increased emergence and rapidly growing prevalence and burden of RVF in Rwanda [3,31,35]. The trajectory of RVF in Rwanda exhibits a complex interplay between ecological factors, the dynamics of human and animal populations, climate change, and agricultural practices, similar to what has been observed in South Africa [42]. The majority of RVF outbreaks have been concentrated in the Eastern Province, which borders Tanzania and Uganda; RVF is endemic in both of these countries, and they frequently report disease outbreaks [3,30,31,35,40]. This geographical clustering suggests the involvement of cross-border transmission, which might be facilitated by the free movement of human and animal populations in the region [30,35,40,43]. 

The unique ecological characteristics of the Eastern Province contribute significantly to the persistence of RVF in the region. The presence of Akagera National Park, a large wetland area that harbors various wildlife species, including buffaloes, might play a crucial role in sustaining the virus’ transmission in the area through a sylvatic cycle [7]. The high seroprevalence of the RVFV that was detected in livestock grazing near the park further supports this theory, which further states that close contact and interactions between wildlife, domestic animals, and humans in the presence of competent mosquito vectors in swampy environments sustain and enhance the local circulation of the RVFV [44,45]. Furthermore, such an environment is optimal for the growth of mosquito vector populations and increased contact between different host species, which represents a typical ecosystem for cross-species sharing and the transmission of zoonotic viruses, including the RVFV [46]. 

Moreover, the climatic conditions in the Eastern Province add another layer of complexity to RVF outbreaks. The region is characterized by an arid climate with sudden metrological shifts, such as the drought followed by flash floods observed in March 2022 in the Nyagatare district [18,47]. Such characteristics enforce changes in the daily practices and movements of human and animal populations, which might increase their vulnerability to RVF’s outbreaks. Other areas in East Africa with similar environmental conditions are challenged with frequent outbreaks of various arboviruses, including RVF, Chikungunya, Crimean–Congo hemorrhagic fever (CCHF), and dengue [8,25,48,49]. These environmental changes likely create optimal conditions for the hatching of infected desiccation-tolerant eggs of the floodwater mosquito *Aedes vexan*, which is a competent vector of the RVFV. Furthermore, due to its unique biology and ability to transovarially transmit the RVFV, it plays a major role in sustaining RVFV transmission; hence, it is known as the initial vector of RVFV outbreaks [6,50,51]. The subsequent surge in mosquito populations in these swampy areas can amplify transmission among wildlife [52]. Furthermore, humans and animals visiting these areas for seasonal grazing and other temporal activities might act like bridging hosts that enhance spillover events, bridging the sylvatic and urban cycles of RVF transmission [7,8]. Additionally, the unique characteristics of the Eastern Province in Rwanda include the fact that it has open international borders with Tanzania and Uganda. These borders are featured by heavy dynamics of human and animal populations on both sides. Considering that RVF is heavily endemic in both Tanzania and Uganda, these population dynamics intensify the vulnerability of this province to multiple outbreaks and a growing burden of RVF. This is supported by the genomics analysis of RVFV isolates involved in the disease outbreaks in 2018 and 2022 in Rwanda, which were confirmed to have originated from a clade of the RVFV that is mainly endemic to and frequently outbreaking in Kenya, Tanzania, and Uganda [35]. 

The Southern Province, while less exposed to extreme climate conditions than the Eastern Province, also experiences metrological conditions that favor mosquito vector population growth due to optimal temperatures and humidity [31]. The presence of large populations of cattle and goats in both the Southern Province and the Eastern Province intensifies the risk of frequent RVF outbreaks, which might be of particular concern for the neighboring country, namely Burundi, which had not reported any cases of RVF until April 2022 [31]. In particular, it is well established that climate change is driving the spread of invasive disease vectors of arboviruses in Africa, increasing the risk and vulnerability of countries with inadequate preparedness and prevention capacities, including diagnosis and surveillance systems [18,26,53,54].

In contrast, the Northern Province and the Western Province of Rwanda have reported fewer RVF cases and a later emergence of the virus, suggesting that they have been less affected by the outbreaks thus far. Movements of cattle from the eastern and southern provinces toward the west and the Democratic Republic of Congo, and vice versa, are likely contributing to the spread of the RVFV in these areas. However, it is essential to remain vigilant, as the potential for the RVFV to become endemic in Rwanda is dire, which calls for strengthening the country’s pandemic preparedness and global health security. 

Nevertheless, it is important to consider that Rwanda is surrounded by countries that are heavily endemic with RVF, mainly including the Democratic Republic of Congo (DRC), Uganda, and Tanzania. However, due to the connectivity of East African countries, as naturally expected, the prevalence and burden of RVF are rapidly growing throughout most East African countries altogether [9]. This is highlighted by the increased frequency and size of RVF outbreaks in the region [9,55,56,57,58,59]. RVF and other arboviral diseases, including Chikungunya and dengue, more or less have the same transmission patterns, and they are commonly affected by similar factors, like climate change and globalization [25,53,60,61]. Therefore, the country should consider establishing a national integrated policy, strategic plan, and surveillance system for monitoring, preparing for, and preventing the introduction of arboviruses and further outbreaks [4]. 

Considering this, farming plays a crucial role in Rwanda’s economy, and the significant losses experienced during outbreaks of these diseases can have far-reaching consequences for livelihoods and food security in the country [31]. Therefore, an effective, national, integrated One Health strategy must include direct collaboration with the agricultural and food authorities in the country.

The lessons learned from these outbreaks suggest that there is an urgent need to enhance preparedness and prevention measures, including improving the country’s diagnostic capacity, monitoring disease dynamics, improving rapid response capabilities, and carrying out public awareness campaigns [62,63]. In particular, RVF is on the WHO’s, CEPI’s, and GAVI’s lists for diseases that might cause the next pandemic [13,14,15]. Therefore, an effective preparedness, prevention, and response framework for zoonotic diseases such as RVF should follow a multisectoral One Health strategy to strengthen global health security through timely data sharing in health emergencies, multisector cooperation, and cross-country coordination [62,63,64,65,66]. The adoption of climate-smart agricultural practices alongside the development and implementation of cost-effective vaccination programs for livestock could play a pivotal role in mitigating the impacts of RVF. 

The growing prevalence and burden of RVF in Rwanda, along with its growing host range and geographical distribution, are putting serious pressure on health systems for humans, animals, and the environment in the country [3,31]. The growing burden and prevalence of RVF in Rwanda calls for effective change to health policies and strategic planning; more importantly, it shows the need for innovative sustainable solutions, such as the integration of genomics tools in the routine surveillance of pathogens and disease vectors. Health authorities in the country should consider the implementation of genomically enhanced xenosurveillance for the early cost-effective detection of pathogens, including RVFV circulation among human, animal, and vector populations [66,67,68,69]. This approach offers a powerful, robust, and cost-effective intervention for improving the early detection and timely surveillance of changes in pathogen and vector compositions and dynamics and host-specific exposure. The continuous generation of such up-to-date evidence will guide policymaking, strategic planning, and the implementation of cost-effective and sustainable preparedness, prevention, and control measures, including host-specific targeted interventions. This will definitely reduce the morbidity, mortality, and societal cost of disease outbreaks in the country [70,71]. Most arboviral disease infections, including RVFV infections, are frequently misdiagnosed, and patients are often mistakenly treated for malaria in malaria-endemic countries, which results in the under-detection and under-reporting of specific diseases [28,72,73]. 

However, the current unique state of RVF in Rwanda offers a suitable opportunity to involve the country in the development and evaluation of and clinical trials for progressing the currently underdevelopment RVF vaccine for humans [74,75]. Actually, considering that Rwanda championed the cost-effective and timely deployment of an RVF vaccine for livestock throughout the country through the innovative use of drones, it might be a suitable place for implementing the planned preclinical studies and clinical trials for a human RVF vaccine [38]. This would create an optimal opportunity not only to explore the effectiveness and safety of the vaccine but also to determine the most cost-effective vaccination mechanisms to protect both human and animal populations from RVF. 

Overall, risk factors like changes in climate, land use, land cover, and the local composition of competent disease vectors, increased cross-border movement of human and animal populations, and unplanned urbanization might be driving forces for the development of these outbreaks [2,8,18,19,25,26,72]. The involvement of these factors is indicated by the recent co-emergence and development of several outbreaks in Rwanda, including mpox, Marburg virus diseases, and an increased burden of malaria and fungal infections, as well as the growing prevalence of antimicrobial resistance (AMR) [22,24,76,77,78,79,80,81]. This underscores the need for a holistic approach through implementing an integrated One Health disease surveillance and response systems that targets diseases of importance to public health in the country, with cost-effective prevention and control interventions [23,66,82,83,84,85,86]. This will improve the public health of humans, as well as animal and environmental health, strengthening global health security, along with food security and socioeconomic stability and growth [36,83,87]. 

There is growing evidence of the rapid spread of the invasive disease vector *Anopheles stephensi* across Africa, and it has been suggested to play a role in the transmission of the RVFV, the Chikungunya virus, and the O’nyong-nyong virus [54,88,89,90]. Similarly, the spread of the competent arboviral diseases vector *Aedes albopictus* is playing a leading role in the growing burden of arboviruses, including RVF, Chikungunya, and dengue, in the region [53]. This stresses the need for a major shift in the national integrated program for disease vector surveillance and control to incorporate the use of genomics and molecular tools in routine surveillance to guide the implementation of an integrated, cost-effective, and sustainable vector management strategy using environmentally friendly strategies and interventions [26,27,53,88,91].

One of the main limitations of this review is the shortage of reports about RVF in Rwanda, particularly the severe lack of information about disease vectors in the country. This underscores the fact that this disease is highly understudied in this country. This, in turn, has resulted in persistent gaps in knowledge about RVF in the country; therefore, investment in research is essential to gather further evidence about the disease’s ecology, host range, and vector composition, as well as the risk factors and associated socioeconomic burden of the disease in this country [4]. Strengthening the research capacity in the country with a focus on emerging diseases will support the development and implementation of evidence-based policymaking, strategic planning, prioritization, resource mobilization, and the implementation of cost-effective preventive and control measures. Knowledge dissemination about RVF outbreaks, symptoms, transmission modes, and preventive and control strategies will empower farmers and communities at risk to take the lead in implementing community-led, cost-effective, and sustainable prevention and control strategies, including increasing preparedness, the early detection of, and timely responses to potential outbreaks, thereby safeguarding public and global health in the country and the region [62]. Such timely sharing of information and transparent public communication will restore public trust in the health system and enhance the implementation of International Health Regulations (IHRs 2005), including multisectoral collaboration and cross-country coordination [22,23,83,84,92].

The well-established national immunization program in the country, which has recently supported the experimental use of a Marburg virus vaccine in response to the recent outbreak [24], means that it is capable of leading strategic investments in investigating the cost-effectiveness of introducing RVF vaccinations for human [74,75]. However, this will require the country to collaborate with global stakeholders such as the WHO, GAVI, and CEPI to conduct clinical trials to assess the cost-effectiveness of vaccinating people at risk of the disease and their livestock in the country against RVF, as well as investigating the most cost-effective vaccination strategy [13,74,93]. Such research is warranted to produce powerful evidence about the best practices and most cost-effective vaccination mechanisms against RVF to prevent the next pandemic [13,14,17,94,95,96,97]. The evidence that will be generated from this study will inform global health policies and strategies to strengthen global health security, food safety and security, and socioeconomic stability and growth and eventually lead to achieving the associated UN Sustainable Developmental Goals (SDGs) in Africa [13,14,98,99,100,101].

Considering that RVF is a climate change-sensitive disease and it is highly affected by changes in land use and land cover, human and animal population dynamics and movements, and vector composition, it will be helpful for countries affected and at risk of RVF to establish an integrated One Health surveillance and response system. Such a system should incorporate and synthesize data and information from all sectors, including metrological data as a main predictor for RVF outbreaks [52,100,102]. Supporting this system with xenosurveillance will substantially reduce the probability of disease outbreaks by capitalizing on early preparedness and prevention interventions rather than late and more costly responses.

## 5. Conclusions

In conclusion, the historical context of RVF outbreaks in Rwanda from 2010 to 2022 reveals a concerning trend that necessitates immediate improvements in disease vector surveillance and control programs by integrating the use of genomics and molecular tools into routine surveillance for the identification of disease vectors. Considering the variation in vector bionomics and susceptibility to different vector control interventions, the accurate identification and characterization of vectors will inform policymaking and guide the planning, development, and implementation of cost-effective, environmentally friendly, and sustainable interventions. By prioritizing research, surveillance, and education, health authorities in Rwanda can work towards effectively managing RVF and mitigating its adverse impacts on human, animal, and environmental health, food security, and socioeconomic stability and growth in the country. The geographical, ecological, and climatic factors influencing the transmission of RVF in Rwanda highlight the need for a multisectoral, region-sensitive, integrated One Health strategy that prioritizes strengthening preparedness and prevention strategies while improving case management. The country should strengthen their early preparedness and prevention strategies by improving diagnosis and surveillance through the implementation of genomically enhanced xenosurveillance for monitoring the dynamics of disease vectors and pathogens, including the RVFV. Additionally, the country should integrate the use of advanced technology such AI-powered drones for vector surveillance and control as well as the delivery of vaccines, and samples to regional laboratories for confirmation tests. This will enhance the country’s national pandemic preparedness, prevention, and response framework with a climate change-friendly approach. 

## Figures and Tables

**Table 1:** Summary of Rift Valley fever outbreaks and epidemiology in Rwanda up to December 2024.

Year	Host	Prevalence	Deaths	Abortions	Location	Diagnosis	Report
2010–2012	Cattle	29.4 (5/17)	NA	NA	NA	Seroprevalence	[41]
2010–2012	Goat	42.9 (3/7)	NA	NA	NA	Seroprevalence	[41]
2012–2013	Livestock	100.0 (30/30)	NA	NA	NA	RT-PCR	[34]
2011–2013	Bovine	16.8 (100/595)	NA	NA	Kirehe, Bugesera, Kamonyi, Gakenke, Ngoma, and Nyagatare	Seroprevalence	[30]
2014–2018	Livestock	100.0 (94/94)	NA	NA	NA	RT-PCR	[39]
2018	Cattle	28.0 (44/157)	NA	NA	Countrywide	RT-PCR	[40]
2018	Goat	42.9 (12/28)	NA	NA	Countrywide	RT-PCR	[40]
2022	Cattle	100.0 (1285/1285)	516	1254	Countrywide	RT-PCR	[31]
2022	Goat	100.0 (34/34)			Countrywide	RT-PCR	[31]
2022	Sheep	100.0 (23/23)			Countrywide	RT-PCR	[31]
2022	Human	100.0 (27/27)	0	NA	Countrywide	RT-PCR	[29]
2022	Human	100.0 (173/173)	22	NA	Countrywide	RT-PCR	[3]
